# The Cell Collective: Toward an open and collaborative approach to systems biology

**DOI:** 10.1186/1752-0509-6-96

**Published:** 2012-08-07

**Authors:** Tomáš Helikar, Bryan Kowal, Sean McClenathan, Mitchell Bruckner, Thaine Rowley, Alex Madrahimov, Ben Wicks, Manish Shrestha, Kahani Limbu, Jim A Rogers

**Affiliations:** 1 Department of Mathematics, University of Nebraska at Omaha, Omaha, NE, USA; 2 College of Information Science and Technology, University of Nebraska at Omaha, Omaha, NE, USA; 3 Department of Pathology and Microbiology, University of Nebraska Medical Center, Omaha, NE, USA

## Abstract

**Background:**

Despite decades of new discoveries in biomedical research, the overwhelming complexity of cells has been a significant barrier to a fundamental understanding of how cells work as a whole. As such, the holistic study of biochemical pathways requires computer modeling. Due to the complexity of cells, it is not feasible for one person or group to model the cell in its entirety.

**Results:**

The Cell Collective is a platform that allows the world-wide scientific community to create these models collectively. Its interface enables users to build and use models without specifying any mathematical equations or computer code - addressing one of the major hurdles with computational research. In addition, this platform allows scientists to simulate and analyze the models in real-time on the web, including the ability to simulate loss/gain of function and test what-if scenarios in real time.

**Conclusions:**

The Cell Collective is a web-based platform that enables laboratory scientists from across the globe to collaboratively build large-scale models of various biological processes, and simulate/analyze them in real time. In this manuscript, we show examples of its application to a large-scale model of signal transduction.

## Background

The immense complexity in biological structures and processes such as intracellular signal transduction networks is one of the obstacles to fully understanding how these systems function. As understanding of these biochemical pathways increases, it is clear that they form networks of astonishing complexity and diversity. This means that the complex pathways involved in regulation of one area of the cell (so complex that a researcher could spend their entire career working in that area alone) are so interconnected to other, equally complex areas that all of the different pathway systems must be studied together, as a whole, if any of the individual components are to be understood. However, the large scale and minute intricacy of each of the individual networks makes it difficult for cell biologists or biochemists working in one area of a cell’s biochemistry to be aware of, let alone relate their results to, findings obtained from the various different areas. So how will all of these individually complex systems be possible to study in an integrated biochemical “mega-system?”

In order to address this problem, the concept of systems biology study has emerged [1-8]. However, with i) data being generated by laboratory scientists at a staggering rate in the course of studying the individual systems, ii) the fact that these individual systems are so complicated that scientists rarely have detailed knowledge about areas outside those that they study, there is a huge impediment to implementing a systems approach in cellular biochemistry, and iii) for laboratory scientists to fully embrace systems biology computational tools must lend themselves to usage without requiring advanced mathematical entry or programming.

Several significant advancements in the systems biology field have been made as a response to the sea of data being generated at ever increasing rates. For example, in the area of biochemical signal transduction, several community-based projects to organize information about signal transduction systems such as the Alliance for Cellular Signaling [9], the former Signal Transduction Knowledge Environment [10], UniProt [11], or the WikiPathways project [12] have been created. These resources provide a way to organize and store important laboratory-generated data and information such as gene sequences, protein characteristics, interaction partners, etc.; these are then easily accessible via the Internet to the scientific community. Building on these resources and advancements has been the development of tools to visualize and analyze these data and, specifically, the entities that make up the complex, network-like structures of biological processes. Amongst the most widely used tools to visualize biological networks is the open-source software, Cytoscape [13].

The information contained in the above database resources (and visualized via Cytoscape) is limited in that it is mostly static; biological systems however are dynamic in nature. Hence to fully understand the underlying mechanisms (and those of corresponding diseases), the dynamics of these processes need to be considered.

Computational modeling and simulation has been successfully adopted in a number of fields to dramatically reduce development costs. The use of these modern tools to organize and probe biological structure and function has a high potential to provide the basis for new breakthroughs in both basic understanding of cell function and the development of disease therapies. The ability to observe the actual dynamics of large scale biological systems increases the probability that, out of the tens of thousands of combinations of interactions, unexpected points of intervention might be deciphered. The Cell Collective aims at providing an environment and resource where the biomedical community, as a whole, can more effectively bring these exciting new computational approaches to bear on cellular systems. The integration of computational and laboratory research has the potential to lead to improved understanding of biological processes, mechanisms of disease, and drug development.

If a “systems approach” is to be successful, then there must be a “system” into which the thousands of laboratory scientists all over the world can incorporate their detailed local knowledge of the pathways to create a global model of biochemical pathways. With such a systems platform, all local information would be far more accurate if laboratory scientists would contribute their specialized expertise into a system that enables the integration of the currently dispersed knowledge. Hence, a collaborative modeling platform has the potential to substantially impact and move forward biomedical research.

This is precisely the purpose of The Cell Collective. The Cell Collective is an environment to model biological processes. The platform allows scientists to deposit and track dynamical information about biological processes and integrate and interrogate this knowledge in the context of the biological process as a whole. Laboratory scientists can directly simulate large-scale models in real time to not only help test and form new hypotheses for their laboratory research, but also to make research more easily reproducible (through sharing their models with collaborators). Furthermore, the creation and simulation of models in The Cell Collective doesn’t require direct use of mathematics or programming – a substantial advancement in the field [14]; this tool has been developed to bring modeling into the hands of mainstream laboratory scientists.

### The role of The Cell Collective in the current landscape of systems biology technology

As a result of the constant flow of data from laboratories, the success of biomedical research relies now, more than ever, on computational and computer technologies. While a number of different technologies have already been developed and succeeded in their purpose, The Cell Collective further builds on the successes of these efforts to provide a novel technology to exploit the full potential of systems biology. In this section, a discussion of some of these technologies follows. Note that, the following is not an extensive review, rather we aim to illustrate how The Cell Collective fits within the landscape of systems biology resources. For better understanding, these resources have been categorized according to their function. 

A) Biological databases (as mentioned in the Background section, Alliance for Cellular Signaling [9], STKE [10], UniProt [11], the WikiPathways project [12], KEGG [15], UniProt [16], Reactome [17], Pathway Commons [18], etc.) were developed as one of the first steps to deal with the sea of biological data being produced with high-throughput technologies. The information contained in these biological databases focuses on static cell “parts lists.” In other words, the data focuses on the description of the individual entities rather than the dynamical relationship between the individual parts. Conversely, The Cell Collective, and specifically its Knowledge Base component (discussed in the Results section) extends static knowledge and data into dynamical models; hence the information contained in the Knowledge Base (which is purely qualitative) is dynamical in nature; it takes into account the dynamical relationship between all of the interacting partners.

B) Software for dynamical models (which employ mathematical frameworks similar to the ones used in The Cell Collective – i.e., rule-based formalisms) also already exist (e.g., GINsim [19], BooleanNet [20], CellNetOptimizer [21], or BoolNet [22]). These tools have been built and used mainly for individual groups to study networks of a confined size. They also rely on the users’ training in computer programming and/or mathematics (and hence are first and foremost tools developed for modelers); this makes it difficult for laboratory scientists to incorporate these tools into their experimental studies. The Cell Collective provides a novel tool in the area of large-scale, whole cell models, while extending the use of computational modeling to laboratory scientists.

C) Model repositories such as the CellML repository [23] or the BioModels Database provide a central location to store models developed by the community. These models are then available to others for download and further analyses using other tools. The BioModels Database is primarily a model repository, however, it does provide simulation capabilities via the JWS simulator [24]. In addition, the PathCase systems biology tool [25,26] provides a central place for kinetic models from the BioModels Database and KEGG pathways to be queried, visualized, and simulated side-by-side. Similar to these resources, The Cell Collective provides the first repository (with simulation capabilities) for models based on a qualitative mathematical formalism.

D) Model exchange standards such as the Systems Biology Markup Language (SBML, [27,28]) or CellML [29] make it easier for models to be exchanged between different groups and simulated/analyzed by different simulation tools. For example, when a research group wants to simulate a model deposited to the BioModels Database, the model’s description in SBML or CellML ensures that the model truly corresponds to the same model used by a different group, and hence the generated data can be easily reproduced. While users can share their models with other users of The Cell Collective directly, without the need to import/export model files, the platform currently provides SBML export features based on the most recent version of SBML L3 qualitative package [30].

E) Visualization and analysis tools for static interaction networks, such as the aforementioned Cytoscape [13], but also others including VisANT [31] or Gephi (http://gephi.org), have been used extensively to visualize and analyze the graph properties of networks of various types and sizes. As a complement to existing graph analyses, The Cell Collective deals with dynamical models – ones that can be put in motion via computer simulations – and hence focuses on the visualization of the dynamics of these models via simulations, and susbsequent analyses (e.g., input-output relationships). Together, The Cell Collective is a platform that not only provides a unique combination of successful systems biology and modeling approaches, but also offers significant innovations to these technologies. In this manuscript, discussed are the various components and features of the platform, and exemplified on a previously published large-scale network model of signal transduction [32].

## Implementation

The Cell Collective is a server-based software implemented in Java and powered by MySQL database. The simulation engine is based on ChemChains which was implemented in C++ [33]. The user interface of The Cell Collective was implemented primarily using JavaServer Faces (http://www.javaserverfaces.org) and Primefaces (http://www.primefaces.org).

### Computational framework and simulations

Models in The Cell Collective are based on a qualitative, rule-based mathematical framework. In this framework, each species can assume either an active or inactive state. Which state a species assumes at any given time point depends on a set of rules that take into account the activation state of all immediate upstream regulators.

**Figure 1:**
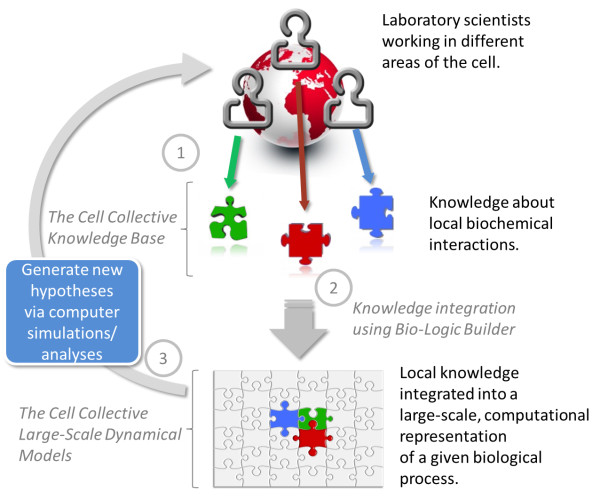
**Construction of models prior to their simulations via built-in ChemChains.** The bio-logic for each species (defined by users) is converted (automatically) to a mathematical (Boolean) expression. Each species’ expression is encoded to a C++ file, and all files are subsequently compiled into a single dynamic library (.so file) which can be read and executed by ChemChains for simulations.

The Bio-Logic Builder provides the user interface for users to enter qualitative information about the regulatory mechanism of each species in a model, and subsequently converts this information into an appropriate mathematical (algebraic) expression (manuscript submitted). Before the simulation engine (ChemChains) can simulate a model, the mathematical expressions of individual species are converted into C++ (.cpp) files, which are subsequently compiled into a single dynamical library (.so file). This dynamical library encodes the entire model which is subsequently simulated by ChemChains (see Figure 1).

Though a discrete (active/inactive) mathematical framework is used to represent the modeled biological processes, ChemChains has been developed to enable simulations of discrete models while using continuous input/output data. In general, the activity levels of the models’ individual constituents is measured as %ON. Depending on the context of the biological process being simulated, this measure corresponds, for example, to concentration or the fraction of biological species being active at any given time.

In the case of real-time simulations, %ON of a species represents its moving average activity, and is calculated as the fraction of the active/inactive states over a sliding window. For simulations using the Dynamical Analysis feature, the activity levels of the individual species (or %ON) also corresponds to the ratio of active/inactive states, but is calculated once the dynamics of the model settle in a steady behavior (or an attractor as described in great detail in [33]). In both the real time simulations and dynamical analysis, %ON is used as a semi-quantitative way to measure the dynamics of the modeled biological processes.

### Simulation performace

We analyzed the perfomance of individual simulations for randomly generated models of different sizes and different complexities (in terms of network connectivity). Specifically, we considered models with 10, 100, 500, and 1,000 nodes and network connectivities of 2, 5, 10, 20, and 100. Note that for biological application, relatively small (low single digit) connectivity is most realistic [32,34,35]. As can be seen Table 1, simulations in The Cell Collective are relatively efficient as the required computational resources are in a linear relationship with the increasing parameters of the generated networks.

**Table 1:** Simulation performance for models with ranging complexity


**# of nodes/connectivity**	**2**	**5**	**10**	**20**	**100**
10	0.88s	0.94s	0.85s	0.99s	0.93s
100	4.82s	4.98s	5.55s	5.95s	9.8s
500	26.99s	29.42s	32.11s	37.31s	68.73s
1,000	60.89s	64.61s	70.95s	79.59s	149.34s

Simulations consisted of 10,000 time steps and were performed on a computer with a single core, 2GHz processor and 2GB of RAM.

## Results and discussion

**Figure 2:**
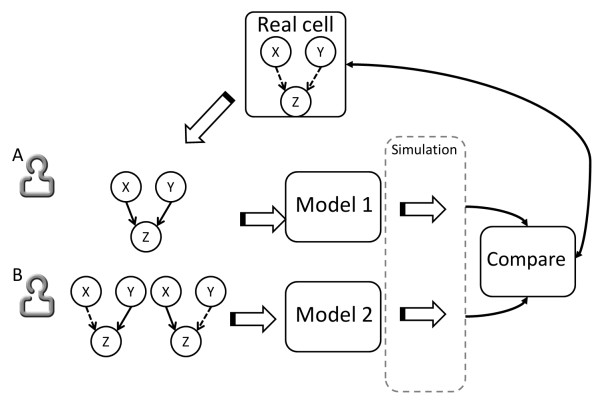
Overview of the flow of knowledge about biological processes, and the role of The Cell Collective in integrating and understanding this knowledge in the context of the biological processes as a whole.

The Cell Collective is a web-based platform (accessible at http://www.thecellcollective.org) in which laboratory scientists can collaboratively build mathematical models of biological processes by utilizing existing laboratory data, and subsequently simulate the models to further guide their laboratory experiments. Conceptually, the platform can be broken up into three parts (Figure 2) that form the basis for the core functionality of the software: 1) integrated Knowledge Base of protein dynamics generated from laboratory research in a single repository, 2) integration of this knowledge into mathematical representation that allows visualization of the dynamics of the data (i.e., put it in motion via simulations), and 3) simulations and analyses of the model dynamics. As can also be seen in the figure, these three parts form a loop that is closed by laboratory experimentation. The first model in The Cell Collective (available in for all users to simulate and build upon) is one of the largest models of intracellular signal transduction [32]. Features available in the current version of The Cell Collective are described in more detail in the following sections.

### Knowledge Base of interaction dynamics

When laboratory scientists produce new results, for example regarding the role of one protein interacting with another protein, these results are usually published along with thousands of other results generated by the scientific community. The publication of individual results in isolation means that separate findings are not necessarily absorbed, verified, analyzed, and integrated into the existing knowledge. With the invention of various high-throughput technologies, the gap between the amount of knowledge produced and the ability of the scientific community to fully utilize this knowledge has grown [36].

The first major component of The Cell Collective (as highlighted in Figure 2) is a Knowledge Base which enables laboratory scientists to contribute to the integration of knowledge about individual biological processes at the most local level which includes, for example, the identification of direct protein-protein interactions. However, the goal of The Cell Collective is not to duplicate other well-established resources by providing extensive parts lists that make up various biological processes and cells. Instead, the aim of the platform is to extend static knowledge and data into dynamical models; hence the information provided in the Knowledge Base needs to be *dynamical* in nature. This means that the information (which is purely qualitative – see the Methods section) contained in The Cell Collective Knowledge Base takes into account the dynamical relationship between all of the interacting partners. For example, let’s assume, there are two positive regulators ( *X* and *Y *) of a hypothetical species *Z*. While in the context of a parts list, information about the above species and interactions would be sufficient, in order to abstract the biological process to a dynamical model, one needs to know the dynamical relationship between the interacting partners. For instance, are both *X* and *Y * necessary for the activation, or is either one of them sufficient to activate *Z*? This is the type of information that is used to construct dynamical models in The Cell Collective.

**Figure 3:**
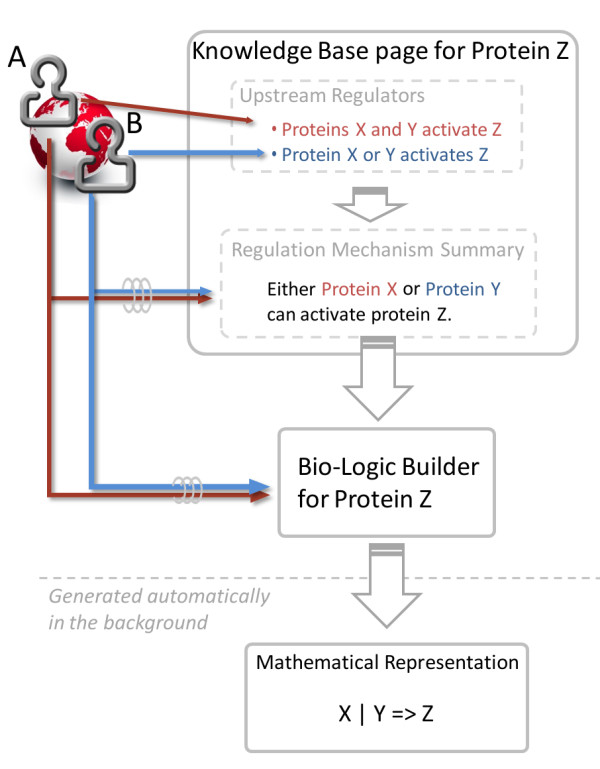
**Integration of laboratory results via modeling.** The different relationships between hypothetical interactions of *X* and *Y * with *Z* as discovered by scientists *A* and *B*. Solid lines depict the necessity of the interaction for species *Z* to be activated, whereas dashed lines correspond the optional nature of the interaction. Because scientist *B*’s results suggest an “OR” relationship between the regulators, there are two graphical representations of *Z*’s regulatory mechanism.

Based on a widely known wiki-like concept, the Knowledge Base module of the platform was developed to allow laboratory scientists to contribute – collaboratively – their knowledge to the complete regulatory mechanisms of individual biological species. Because all of the regulatory information forms the basis of the modeled biological/biochemical process, and hence has to be correct for the model to exhibit similar behaviors as seen in the laboratory, this process of aggregating all known information about a species into one place can also serve as a mechanism to identify possible contradictions or holes in the current knowledge about the regulatory mechanism of a particular species. Using the previous hypothetical example, let’s assume laboratory scientist *A* discovers that proteins *X* and *Y * are **both** necessary to activate species *Z*, but scientist *B*’s laboratory results suggest **either** protein *X***or***Y * can sufficiently activate *Z* (Figure 3). The process of integrating all known information on species *Z* becomes crucial in discovering such discrepancies (or additional missing information), which may have not been found otherwise. Because the goal of The Cell Collective is to also integrate this information into dynamical models, simulations of the large-scale model (which might have hundreds or thousands of additional components in it) can suggest whose data is more likely to be correct. Assume that scientist *A* adds his information into the model and the model exhibits phenomena similar to the ones seen in the laboratory, whereas when the model is built with the data from scientist *B*’s experiments, the simulation dynamics of the overall model fails to resemble the known actions of the real system. In such a case, new laboratory experiments would be warranted, with a potential to produce more insights into the regulatory mechanism of protein *Z* (Figure 3).

The sea of biological information has made it difficult for the data to be verified on such an integrated basis. We fully understand how some of the most complex biological systems work only when the experimental data is re-integrated into and seen in the context of the entire system; a platform for integration of data is exactly what The Cell Collective provides.

#### Dynamical information

Each species in The Cell Collective’s Knowledge Base has a dedicated page where laboratory scientists can directly deposit their knowledge regarding the species’ regulatory mechanisms. While the wiki-like format of the Knowledge Base gives users the ability to input their data in a free form which can be also interactively discussed, each page is structured to help users organize and review their data more efficiently. Because the wiki format is an easy medium for collecting knowledge from a large number of individuals, a number of scientific efforts have successfully adopted a variation of this technology (e.g., [12][32][33][34]).

First, the *Regulation Mechanism Summary* section describes the general mechanism of the activation/deactivation of the species. This section, found at the top of the page of a given species, is most important from a systems perspective as the information therein takes into an account the role of all immediate upstream regulators (see below).

**Figure 4:**
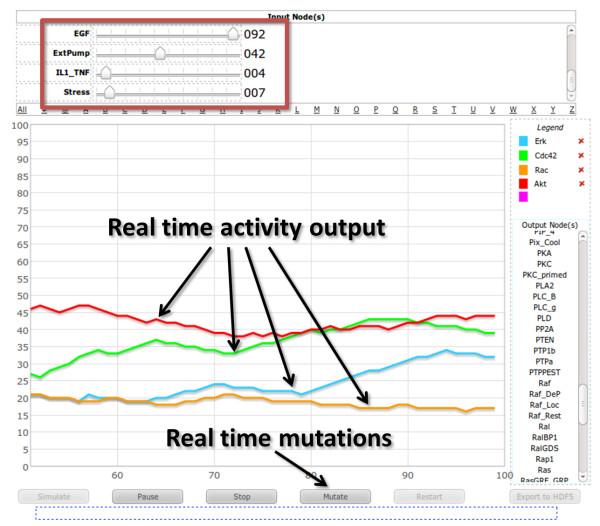
**Visualization of the flow of data generated by laboratory scientists through The Cell Collective Knowledge Base and Bio-Logic Builder.** For example scientists *A* and *B* identify different upstream regulators (protein *X* and *Y *, respectively) of protein *Z*. This knowledge is subsequently recorded in the Upstream Regulators section on the page of protein *Z*. Then both scientists *A* and *B* determine what the relationship is between the two upstream regulators of *Z*. Once the overall regulation mechanism is agreed upon, the scientists use Bio-Logic Builder to add the regulatory mechanism of *Z* to an actual model. The mathematical representation of the species bio-logic is generated in the background, so the user never has to define any mathematical equations nor expressions.

The *Upstream Regulators* section contains the list of key players that have a role in the regulation of the species, as well as any evidence (as found in the laboratory) supporting those roles. Using the earlier example involving the regulatory mechanism of species *Z*, this section would include proteins *X* and *Y * as upstream regulators, and the findings of laboratory scientists *A* and *B* suggesting the role of these regulators in the activation of the species (Figure 4). On the other hand, the Regulation Mechanism Summary section (discussed above) would contain the overall dynamical information as to how *Z* is regulated in the context of both *X* and *Y * (i.e., are both regulators required for the activation, or only one of them?).

*Model-specific Information* section: Because a number of molecular species can be regulated differently based on the type of the cell, this section allows users to enter such cell type-specific information. For example, an intracellular species can be regulated either by different players, or the same players but with different dynamical relationships in, say, a T cell and a mammary epithelial cell. This section enables users to differentiate between the regulatory mechanisms of the species in the two (or more) different types of cells (i.e., models). Hence, this section can be utilized by users to define upstream regulators and the regulation mechanism summary that is specific to users’ different models. For example, the regulation mechanism summary of species *Z* in scientist *A*’s model would describe his findings that both upstream regulators of *Z* are necessary for its activation, whereas scientist *B*’s regulation mechanism summary on wiki page for *Z* would indicate that either one of the upstream regulators can activate *Z* (Figure 4).

Finally, *References* is a section that users can use to record any published works that support information entered in any of the above sections. Users can enter references by simply entering the Pubmed ID (pmid) of the article of interest and The Cell Collective will automatically import all of the bibliographical information about the works.

As a starting point, we have deposited all biological knowledge describing one of the largest dynamical models of signal transduction built and published as part of our previous research [32]. This model consists of around 400 biochemical interactions between 130 species, comprising a number of main signaling pathways such as the Epidermal Growth Factor, Integrin, and G-Protein Coupled Receptor pathways. The dynamical information about the hundreds of local interactions, collected manually from published biochemical literature, is available in the Knowledge Base module. Expert scientists in the field may begin contributing to it, as well as discovering discrepancies and gaps in the biological knowledge that might have been included in the model.

Once the dynamical information about the individual interactions is added in the platform Knowledge Base, the next step is to convert this knowledge into a dynamical model; a discussion on where this piece fits into the overall concept of The Cell Collective follows in the next section.

### Building computational models

While the Knowledge Base component of The Cell Collective serves as the knowledge aggregator for the dynamical regulatory mechanisms of individual biological species, the next step (#2 in Figure 2) is to convert this knowledge into a dynamical computational model that can be simulated and analyzed on the computer.

Perhaps one of the biggest challenges in transforming biological knowledge into a computational model is the conceptual gap between the mathematical and biological sciences. Thus far, the creation of mathematical models has been limited to scientists who are well versed in computer science and mathematics. To address this issue, we have developed Bio-Logic Builder (manuscript submitted), a component of The Cell Collective, which allows laboratory scientists to build computational models based purely on the *logic* of the species’ regulatory mechanisms as discovered in the laboratory.

The step of transforming biological knowledge into its model representation is aided by the information provided in the Knowledge Base component of the software platform (Figure 4). Specifically, as discussed above, the information recorded for the corresponding local interactions by individual scientists amounts to the overall regulation mechanism which represents the blueprint of each species’ *bio-logic*. While the local interactions (concerning a hypothetical protein *Z* in Figure 4) are discovered in the laboratory by individual scientists (for example scientists *A* and *B* as shown in the figure), the species overall regulation mechanism should take into an account all of the local knowledge (and hence should be determined in a collaborative fashion). Bio-Logic Builder was developed in such a way that all that is necessary to construct the computational representation of the regulatory mechanism of each species is the same qualitative data provided in the Knowledge Base component. Scientists define each species’ bio-logic in a modular fashion by simply defining activators and inhibitors (i.e., upstream regulators) of the species of interest, as well as the logical relationship between the upstream regulators (e.g., whether or not a set of activators is required for activation, as discussed in an example above). Because models in The Cell Collective utilize a qualitative, rule-based mathematical framework, no kinetic parameters are necessary to construct the models. (A quick tutorial on how to use the Bio-Logic Builder to construct models is available at http://www.thecellcollective.org)

Once the bio-logic is defined for all species in a given model, *in silico* simulations and analyses can be conducted (step #3 in Figure 2). How this can be done with The Cell Collective is the focus of the next section.

### Simulations and analyses of model dynamics

The idea behind abstracting biological processes as computational models is to be able to visualize the dynamics of these processes on the computer, and to conduct *in silico* experiments that can provide *i)* new insights into laboratory experiments and *ii)* additional basis for theoretical computational research to further elucidate the complexity governing these biological processes. With its simulation and analysis component, The Cell Collective has been designed to provide exactly these features. Specifically, in the current version of the platform, two tools for simulations and analyses (discussed below) are available.

#### Real-time simulations

Perhaps the most unique and novel innovation to computational modeling is the real-time simulation feature in the platform, which allows users to visualize the dynamics of any model interactively and in real time. Similar to the rest of the platform, the simulation features have been designed with simplicity and intuitiveness in mind.

**Figure 5:**
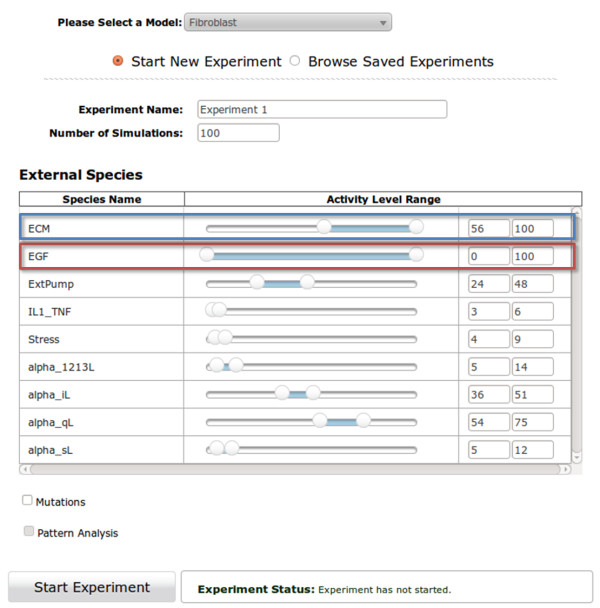
**Screen-shot of a real-time simulation.** Users can change the activity level of the extracellular species via simple sliders (boxed in red). Each tracing in the graph corresponds to an activity level of a species specified in the legend by the user. Any effects of the change of activity of the external species is then reflected in the dynamics of the species’ graph; as the user moves the slider, the activity patterns of the selected species change in real time. In addition, by using the “Mutate” button, users can simulate the effects of gain/loss-of-function mutations on the dynamics the modeled biological process.

All modeled biological/biochemical processes in The Cell Collective, represented by species that make up the internal machinery of the cell, are simulated in external environments which drive the dynamics of the system. In our example of signal transduction, this environment is represented by external species corresponding to various extracellular signals such as growth hormones, stress, etc. Using a simple slider, users can change the amount of each extracellular signal (measured in %ON on a scale of 0 to 100 – see the Methods section for more detail) and visualize the effects of the changes on the dynamics of the cell while the simulation is running. Similarly, users can introduce biological mutations to simulate loss-of-function and gain-of-function experiments while watching the dynamics of the cell change as a result of the mutations. For users’ convenience, real time simulations can be also paused and resumed at any time. Figure 5 shows a screen-shot of the real time simulation tool. A short video demonstration of real time simulations using the previously mentioned large-scale model of signal transduction is also available as a Additional file 1.

#### Dynamic Analysis

Laboratory studies to identify functional relationships between extracellular stimuli and various components of the cell involve a number of experiments that can be both time consuming and resource demanding. For example, a laboratory study [37] that suggests that Akt (a serine/threonine kinase involved in the regulation of a variety of cellular responses such as apoptosis, proliferation, etc.) is activated in response to the Epidermal Growth Factor (EGF), the activity of Akt is measured and compared in untreated cells and cells treated with EGF. Such studies usually involve the construction of a number of protein constructs, cell cultures, assays, etc, amounting to the use of many resources.

While Akt has been known for many years to be activated in response to EGF, there are many areas of the cell that are not as well understood. Laboratory experiments in such areas can be sometimes based on less sound hypotheses that may lead to the waste of many resources. But what if one had the ability to pre-test laboratory hypotheses on the computer, using a computational model, in a matter of minutes? This would allow laboratory scientists to weed out weak hypotheses while focusing on the ones that have a better chance of being proven correct, and hence resulting in more efficient studies.

This is where the Dynamic Analysis simulation feature of The Cell Collective plays an important role. This tool allows users to conduct *in silico* experiments that closely resemble the way laboratory experiments are performed, with the advantage that in these computational studies researchers can perform more simulations and experiments in a much shorter time-frame. For example, models in The Cell Collective can be simulated and their dynamics visualized and analyzed in hundreds or thousands of extracellular environments (as opposed to the limited number of scenarios possible in the laboratory) in a manner of minutes.

**Figure 6:**
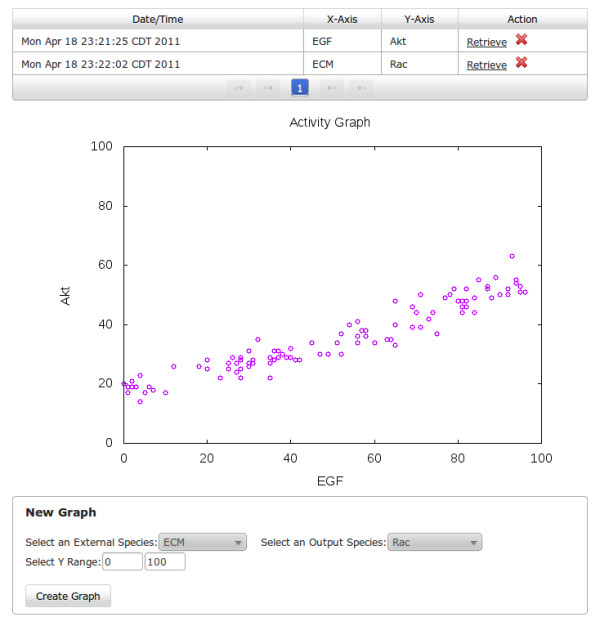
**Dynamical analysis page.** Dynamical analysis page. For each *in silico* experiment, users can use the dual sliders to define the ranges of activity levels of each extracellular species. Users can also set additional properties of the experiment including the number of simulations as well as mutations (gain/loss-of-function).

As an example, we will demonstrate how the software can be used to study the relationship between EGF and Akt. The dynamical analysis studies are done in two parts. First, on the main page of the simulation tool (Figure 6), users define the extracellular environment under which the study will be done. This is analogous to the preparation of cell media in the laboratory. Similar to laboratory experiments with real cells, different studies using computational models (or virtual cells) also require the set up of optimal extracellular conditions. As visualized in the figure, this can be done easily by setting the ranges of the activity (from 0 to 100%) of the individual extracellular (external) species via the dual sliders (or by just typing the activity levels in the appropriate text boxes). Because in this example experiment, we are interested in the effects of EGF on the network model, the activity of EGF (boxed in red) is set to range on the full scale between 0 and 100% ON. On the other hand, the activity ranges of the remaining external species are selected for optimal results based on our previous research [32], and supported by laboratory-generated data. For example, the Extracellular Matrix (ECM) is set to higher activity levels, varying between 56 and 100% (boxed in blue); this corresponds to a biological finding that EGF-induced growth (as well as other cellular processes) is dependent on cell anchorage via ECM [38]. (Note that, from our experience with large-scale models, while optimal conditions should be determined, the simulations and results are not sensitive to exact values.)

While in this example, 100 simulations are performed, users can specify the number of simulations to be run within the study (Figure 6). During each simulation, an activity level for each extracellular species is selected randomly by the software such that the activity falls into the specified range. As a result, the user is able to simulate what would amount to 100 different laboratory experiments, with each experiment corresponding to a different external condition.

**Figure 7:**
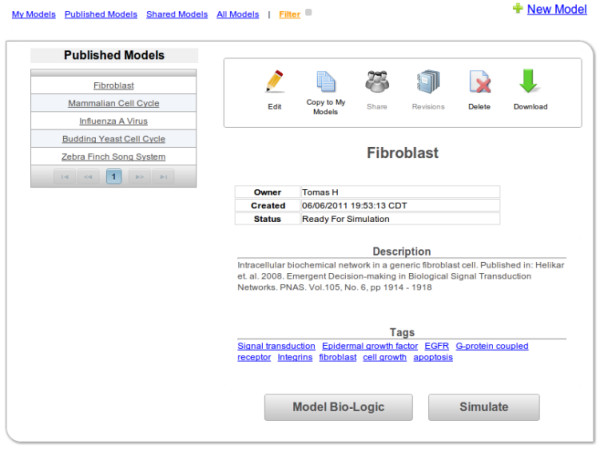
**An example of a dose-response curve visualizing the functional relationship between Akt and EGF.** Users can generate a number of graphs that are saved and can later be retrieved from the table at the top of the page. Generated graphs can also be saved on the computer and used directly in a manuscript.

Once the *in silico* experiment has completed, users can analyze the dynamics of the model. Currently, the Dynamic Analysis tool allows users to generate dose-response curves to investigate qualitative (input-output) relationships between external cellular signals and various components of the model, such as the one between EGF and Akt as visualized in Figure 7. As can be seen in the graph, there is indeed a positive correlation between EGF and Akt, similar to the phenomenon seen in the laboratory. An additional significant advantage of computational experiments using this tool is that users can generate a number of analyses without re-running the entire experiment. For instance, in addition to examining the functional relationship of Akt and growth, one can generate similar dose-response curves for any species in the model using a single 100-simulation experiment. This is done by specifying the appropriate extracellular signal and output species (i.e., any species of interest) from drop-down menus available on the page. On the generated graph, the selected external species is represented on the x-axis whereas the output species is represented on the y-axis. Furthermore, similar to the real time simulation feature, mutations to any of the cellular species can easily be specified which allows users to simulate gain/loss-of-function in an intuitive fashion. In the current version of the software, users can generate the dose-response graphs for all species in the model by selecting the appropriate input-output species. While we are in the course of adding additional means of visualizing the simulation results, users can also download all generated (raw) simulation data, which can subsequently be analyzed by users according to their needs.

The Dynamical Analysis feature can be used not only to generate new hypotheses, but also to test the *correctness* of the model. Because the models are built using local knowledge of the individual interactions, how do we know that all of this local information adds up to a system that represents what is seen in the laboratory? Hence the correctness of the model needs to be tested on global phenomena of the system. The above example demonstrates how the model of signal transduction in a fibroblast cell can be tested to ensure that species associated with apoptosis and growth (such as Akt) appropriately respond to a growth signal (EGF). If, for example, the dose-response curve for Akt and EGF suggested a negative correlation, one would have to go back and investigate which of the local interaction data resulted in the contradictory result.

### Seed models

In addition to the signal transduction model of a fibroblast cell created and previously published by our group [32], as part of our most recent research efforts, we have constructed additional models of the budding yeast cell cycle [39] and host cell infection by Influenza A, including the viral replication cycle (manuscript submitted). We have also re-created a model of ErbB signaling and regulation of the G1/S transition in the cell cycle during breast cancer. This model was initially created by the authors to study trastuzumab resistance and predict possible drug targets in breast cancer [40]. All of these models are now available and published in The Cell Collective, hence available to the scientific community as seed models for further contributions and/or simulations and analyses.

### Collaboration and accessibility

As discussed in the Background section, collaboration amongst laboratory scientists working in different areas of complex biological processes and the accessibility to modeling frameworks is key to new discoveries using the systems approach. These two properties were strictly kept in mind when designing the software, and provide the main framework for The Cell Collective.

First, motivated by this framework was the use a wiki-like format to keep track of the knowledge concerning the dynamical properties of biological process. This framework was also applied to the way users interact with the actual computational models.

**Figure 8:**
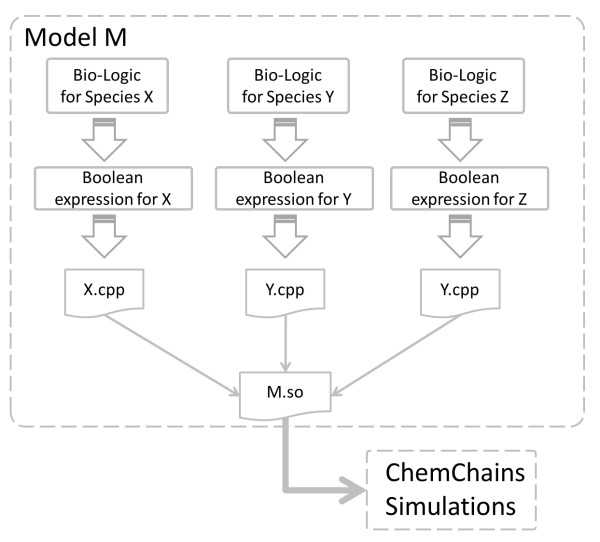
**Main model panel.** The filter in the top left corner allows the user to switch between the different types of models (discussed in text). The majority of the space in the right section of the panel is dedicated to the model’s controls (boxed in) and more general information about the model (e.g., creator and description). Users can also navigate from this panel to the simulation page as well as a page containing all model constituents by using the Simulate and Model Bio-Logic buttons, respectively. As indicated in the right upper corner, users can also initiate the creation of new models from this page.

Perhaps the most important feature in the context of accessibility is the concept of “Published Models” (Figure 8). These models created by the community are freely accessible to all registered users, fostering the idea of open science. All users can view the bio-logic as well as the information in the knowledge base, and perform real time simulations on these models directly. To make changes to these models and see how these modifications affect the dynamics of the model, users can create personal copies of published models. Once a copy of a published model is created, the copy will be available and visible only to the one user until shared under “My Models” as seen in Figure 8. (As mentioned earlier, a number of models are now available under Published Models for all users to access and simulate.)

My Models is a collection of models created by any given user. Users have an additional ability to share and collaborate on any of these models with a select group of colleagues. The degree to which such a collaboration can take place is guided with the choice of three types of permission a user can specify when sharing his/her model. First, models can be shared such that other users can simulate the shared models and view the model’s bio-logic. A second way of model sharing also allows other users to contribute to the models and directly edit them. Finally, models can be also shared so that other users become model administrators and have the same rights as the creator of the model, including the ability to share the model with additional collaborators.

Many biomedical research software tools (especially the commercial ones) tend to limit users in such a way that once the user commits to the tool, it becomes difficult to move their data to a different platform. This is exactly the opposite with The Cell Collective. In addition to being able to share models with any and every user of the platform, features to export models in formats that can work with other modeling tools are also available. In the most recent version, users can export all mathematical expressions for each model (including the available published models) in the form of flat text files as well as SBML (SBML [28]).

Finally, a forum is available as part of The Cell Collective modeling suite. This will afford users additional means of communication with the scientific community as well as with the platform’s development team.

## Conclusions

Because of the inherent size and complexity of biochemical networks, it is extremely difficult for a single person or group to efficiently transfer the vast amount of laboratory data into a mathematical representation; this fact applies to any modeling technique. One way to address this issue is to engage the community of laboratory scientists that have generated these data and, hence, have first-hand knowledge of the local protein-protein regulatory mechanisms. If the community of laboratory scientists had a mechanism by which they could collaborate and contribute their intimate knowledge of local interactions into a large-scale global model, the creation of these models would be greatly enhanced in terms of both size and accuracy. As most laboratory scientists communicate their data in qualitative terms, rule-based models which utilize such qualitative information provide an ideal candidate for that platform.

Although qualitative models do not require an understanding of high level mathematics, it does assume that users dealing with these models are familiar with rule-based (e.g., Boolean) formalisms. At first, this may seem a subtle issue (as most qualitative information generated in laboratories is practically generated and interpreted in Boolean terms; e.g., protein x AND y activate protein z), however, the Boolean truth tables (and expressions) get more complex as the size of the model increases. This complexity effectively creates another challenge in building large-scale models. The Cell Collective and its major component, Bio-Logic Builder (manuscript submitted), aims at bridging this gap by enabling users to create these dynamical models without having to directly interact with the model’s mathematical complexities.

The collaborative nature of The Cell Collective also opens doors to more open and reproducible science. By integrating biological knowledge, currently dispersed across hundreds of scientific papers, scientists will be able to test the integrity of this knowledge in the context of the b/iological processes as a whole. The model building process will make it easier to identify published results that contradict each other, as well as find gaps in current knowledge that may have not been realized. Using a modeling platform such as The Cell Collective has the potential to generate new hypotheses that can be further verified in the laboratory.

Furthermore, the non-technical and easy-to-use nature of building and simulating computational models in The Cell Collective, the platform has a potential as a great educational tool for undergraduate and graduate biology students with diverse mathematical/computer science skills. Rather than studying biochemical pathways presented in current textbooks as “static” and isolated components of the cell, students can easily visualize and start understanding cells as complex, dynamical systems – precisely as is the case with real cells. Large models available in The Cell Collective allow for the instruction of experimental design – because modeled biological processes have (the complex) properties of the real counterparts, students can learn how to design experimental studies, including the concepts of controls. Students can also create simple cellular models and study the dynamical properties of a wide range of molecular subsystems such as positive and negative feedback loops.

We are actively developing new features and making The Cell Collective even more intuitive for users to interact with it. We are also working on implementing a plug-in system to allow the community to be directly involved in the development of additional features.

## Availability and requirements

The Cell Collective is platform independent, and can be accessed through any modern web browser (Firefox and Chromium are recommended). Data made public in The Cell Collective are governed with GNU GPL v.3. The platform is free for academic use.

## Competing interests

The authors declare that they have no competing interests.

## Authors contributions

TH and JAR conceived the platform. TH designed the software and led the development. BK, MS, SM, and KL developed the software. TH, JAR, KB, MS, SM, KL, and AM tested the software. TH and JAR wrote the manuscript. All authors read and approved the final manuscript.

## Supplementary Material

Additional file 1: **Real time simulation example.** Video example of a real time simulation of a large-scale model of intracellular signal transduction. Click here for file
